# “We have a voice, in this too”: The Ethnographic Narratives Detailing the Individual and Structural Challenges of Care Partners of Persons Living with Dementia

**DOI:** 10.1002/alz70858_103945

**Published:** 2025-12-26

**Authors:** Lilcelia A. Williams, Ishan Canty Williams, Quinton D. Cotton, Melita H Terry, Jennifer H Lingler

**Affiliations:** ^1^ Department of Occupational Therapy, University of Pittsburgh, School of Health and Rehabilitation Sciences, Bridgeside Point I 100 Technology Drive, PA, USA; ^2^ Department of Medicine Division of Geriatric Medicine, University of Pittsburgh, School of Medicine, Pittsburgh, PA, USA; ^3^ University of Pittsburgh Alzheimer's Disease Research Center (ADRC), Pittsburgh, PA, USA; ^4^ University of Virginia, Charlottesville, VA, USA; ^5^ University of Pittsburgh, Pittsburgh, PA, USA

## Abstract

**Background:**

Approximately 1.5 million Black Americans serve as care partners for persons living with Alzheimer's disease or related dementias (ADRD) and one out of five Black American adults aged 70 and older are living with ADRD. Black Americans are diagnosed three years later compared to White adults and present with greater symptom severity and at later disease stages. Yet, specific details about the individual and structural challenges experienced by Black Americans serving as care partners of persons living with dementia (PLWD) remain ambiguous.

**Method:**

Guided by the question “What are the specific concerns and perspectives of care partners of PLWD?,” a secondary analysis of ethnographic interview data from the Recruitment Innovations for Diversity Enhancement Study (RIDE). The RIDE study purposively sampled Black American care partners of participants at an NIA‐funded Alzheimer's Disease Research Center (ADRC). Interview data were analyzed using thematic analysis involving line by line coding, category development, and generation of themes across the dataset. Participants (*n* = 11) included spouses (*n* = 6), children (*n* = 2), friends (*n* = 2) and relatives (*n* = 1). Female participants comprised 64% (*n* = 7) of the sample with males accounting for 36% (*n* = 4). The mean participant age was 73 years old (SD±9.94) with a mean education level of 15 years (SD±2.91).

**Result:**

Black American care partners identified individual and structural challenges learning about AD as a condition, its prevalence among Black Americans, AD risk, and protective strategies for reducing risk citing a lack of trust in systems and professionals not actively engaged in the Black community. Further, care partners denoted the burden of caring for a PLWD and reported the need for respite care. Care partners verbalized their interest in maintaining privacy and confidentiality when facing a health challenge. Finally, care partners noted that participation in research was valuable but complicated by experimental and harmful practices previously employed in government and scientific settings.

**Conclusion:**

This analysis is a first step to identifying and meeting the unique needs of a specific community experiencing an increased incidence of ADRD. Our findings show multidimensional and complex challenges experienced by Black American care partners that requires structural changes for culturally sensitive ADRD diagnosis and care.

